# Estimating (Non)Linear Selection on Reaction Norms: A General Framework for Labile Traits

**DOI:** 10.1002/ece3.72298

**Published:** 2025-10-24

**Authors:** Jordan S. Martin, Yimen G. Araya‐Ajoy, Niels J. Dingemanse, Alastair J. Wilson, David F. Westneat

**Affiliations:** ^1^ Evolutionary Ecology of Aquatic Ecosystems Laboratory, Fish Ecology and Evolution Eawag Swiss Federal Institute of Aquatic Science and Technology Dübendorf Switzerland; ^2^ Human Ecology Group, Institute of Evolutionary Medicine University of Zurich Zurich Switzerland; ^3^ Department of Biology Norwegian University of Science and Technology Trondheim Norway; ^4^ Behavioral Ecology Unit, Department of Biology Ludwig Maximilian University of Munich Munich Germany; ^5^ Evolution Group, Centre for Biosciences University of Exeter Exeter UK; ^6^ Department of Biology University of Kentucky Lexington Kentucky USA

**Keywords:** complex trait, flexibility, multivariate, personality, phenotypic evolution, plasticity

## Abstract

Individual reaction norms describe how labile phenotypes vary as a function of organisms' expected trait values (intercepts) and plasticity across environments (slopes), as well as their degree of stochastic phenotypic variability or predictability (residuals). These reaction norms can be estimated empirically using multilevel, mixed‐effects models and play a key role in ecological research on a variety of behavioral, physiological, and morphological traits. Many evolutionary models have also emphasized the importance of understanding reaction norms as a target of selection in heterogeneous and dynamic environments. However, it remains difficult to empirically estimate nonlinear selection on reaction norms, inhibiting robust tests of adaptive theory and accurate predictions of phenotypic evolution. To address this challenge, we propose generalized multilevel models for estimating stabilizing, disruptive, and correlational selection on the reaction norms of labile traits, which can be applied to any repeatedly measured phenotype using a flexible Bayesian framework. Our modeling approach avoids inferential bias by simultaneously accounting for uncertainty in reaction norm parameters and their potentially nonlinear fitness effects. We formally introduce these nonlinear selection models and provide detailed discussion on their interpretation and potential extensions. We then validate their application in a Bayesian framework using simulations. We find that our models facilitate unbiased Bayesian inference across a broad range of effect sizes and desirable power for hypothesis tests with large sample sizes. Coding tutorials are further provided to aid empiricists in applying these models to any phenotype of interest using the Stan probabilistic programming language in R. The proposed modeling framework should, therefore, readily enhance tests of adaptive theory for a variety of labile traits in the wild.

## Graphical abstract

It remains difficult to empirically estimate nonlinear selection on individual reaction norms, inhibiting robust tests of adaptive theory and accurate predictions of phenotypic evolution for labile traits. To address this challenge, we propose generalized multilevel models for estimating stabilizing, disruptive, and correlational selection on the parameters of individual reaction norms, which can be applied to any repeatedly measured phenotype using a flexible Bayesian framework.

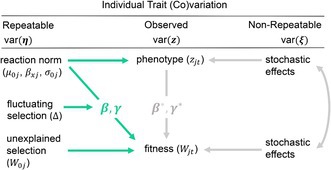

## 1 Introduction

**FIGURE 1:**
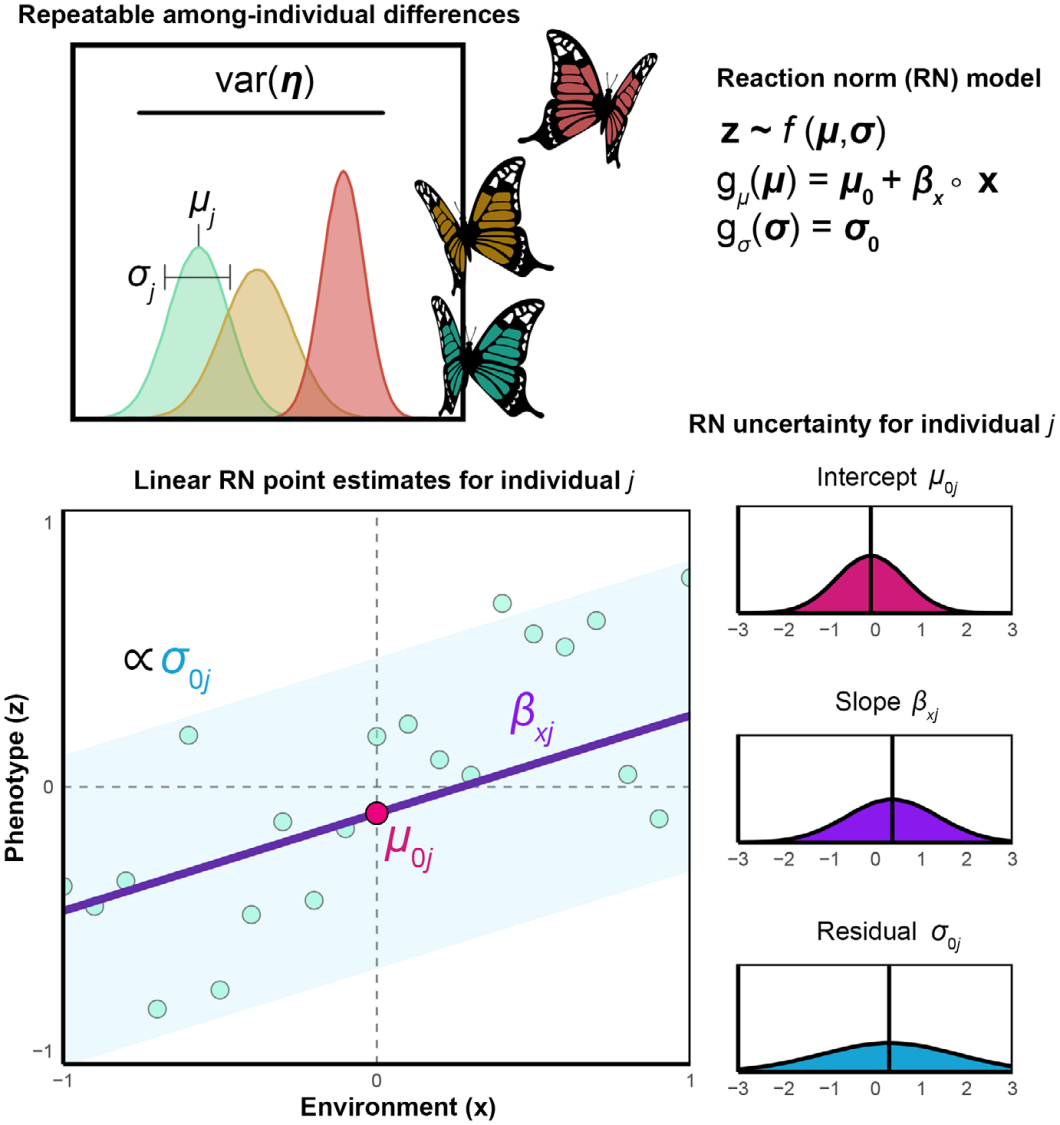
Empirical estimation of individual reaction norms. Repeatable among‐individual differences $\mathrm{var}\left(\eta \right)$ (*top left*; Equations S1 and S2: Appendix S1) in the expected value μ and dispersion σ of observed phenotype **z** can be predicted with a RN model (*top right*) using link functions ***g*** and three (or more) distinct parameters: RN intercept parameters $\mu_{0}$ describing each individual's average phenotype across a mean‐centered environment or in the absence of the environment (i.e., when the environmental state x = 0); RN slope parameters $\beta_{x}$ describing each individual's systematic change in phenotype across environmental states **x**; and RN residual parameters $\sigma_{0}$ reflecting each individual's degree of stochastic variability (or, conversely, their predictability/precision) in phenotype within a given environment. Note that ∘ simply indicates element‐wise multiplication of each environmental state with the corresponding individual slope. See Equation (1) for individual‐level index notation. These parameters will be unknown in empirical research and must be estimated using raw measurements (teal circles) across environmental states (*bottom left*). An example is shown for a simple linear RN with a log‐link on the dispersion of a normal distribution, so that an individual's residual parameter, expressed as a variance on the squared log scale $\text{sqrt}\left(\exp\left(\sigma_{0}+\sigma_{0j}\right)\right)$, is proportional to (∝) the spread of observed residuals on the original data scale, as shown here by a 95% credible interval. Failure to account for uncertainty around point estimates of individual *j*'s RN parameters (*bottom right*) leads to anticonservative inference and increased risk of false positives (Hadfield et al. 2010).

A population will evolve by natural selection whenever heritable variation occurs in fitness‐relevant phenotypes (Darwin 1859). Measuring the fitness consequences of individual differences in highly labile behavioral, physiological, and morphological traits is, therefore, fundamental for explaining their adaptive evolution. Across a variety of phenotypes and taxa, repeatable individual differences have been observed in organisms' average trait values (Bell et al. 2009; Fanson and Biro 2015; Cauchoix et al. 2018) and in their plasticity across environments (Dingemanse et al. 2010; Stamps 2016; Arnold et al. 2019), with some individuals consistently being more or less responsive to environmental change than others. In addition, it is increasingly appreciated that individuals may repeatably differ in their degree of stochastic phenotypic variability within a given environment (Biro and Adriaenssens 2013; Westneat et al. 2013; Mitchell et al. 2021), a phenomenon that has often been ignored in ecological research (Hansen et al. 2006). These individual‐specific patterns reflect distinct but potentially integrated parameters (intercepts, slopes, and within‐individual residuals) of the individual reaction norms (RNs, i.e., state‐dependent functions relating phenotype to environment, Table 1) evolving in a population (Figure 1). RN models provide a highly generalizable, quantitative framework for investigating the evolution and development of labile traits, with broad applications ranging from social behaviors (Dingemanse and Araya‐Ajoy 2015; McNamara and Leimar 2020; Martin et al. 2023) and learning processes (Wright et al. 2022) to thermal performance curves (Svensson et al. 2020) and extended phenotypes (Munar‐Delgado et al. 2023), such as gall size in insect–host plant interactions (Weis and Gorman 1990). Interest in the evolutionary ecology of RNs has grown steadily across a diverse range of fields in recent decades (e.g., Brommer et al. 2012; Strickland et al. 2021; Newediuk et al. 2022), generating methodological innovations for estimating RNs subject to measurement error (e.g., Nussey et al. 2007; Dingemanse and Dochtermann 2013; Gomulkiewicz et al. 2018; O'Dea et al. 2021; Martin and Jaeggi 2022), as well as theoretical models for explaining the selection pressures shaping and maintaining individual variation in RNs within populations (e.g., Wolf and Weissing 2010; Dall and Griffith 2014; Sih et al. 2015; Wright et al. 2019). Attention to RNs has also increased in related fields of inquiry such as personality psychology (Denissen and Penke 2008; Nettle and Penke 2010) and evolutionary anthropology (Jaeggi et al. 2016; Martin et al. 2023).

**TABLE 1:** Notation and terminology.

Term	Symbol	Description
Individual reaction norm (RN)	$f\left(\mu ,\sigma \right)$	A probabilistic function f with parameters predicting the expectation μ and dispersion σ of an individual's phenotype in response to a measurable aspect of the environment.
RN intercept	$\mu_{0},\mu_{0j}$	The expected phenotype in the average environment or in the absence of an environmental factor. Individual RN intercept $\mu_{0j}$ is expressed as a deviation from population RN intercept $\mu_{0}.$
RN slope	$\beta_{x},{\beta_{x}}_{j}$	The expected change in phenotype in response to a measured environment *x*. Individual RN slope $\beta_{\mathrm{xj}}$ is expressed as a deviation from the population average slope $\beta_{x}$.
RN residual	$\sigma_{0},\sigma_{0j}$	The magnitude of stochastic variability in phenotype within a given environment, that is, the inverse of predictability (O'Dea et al. 2021) and precision (Hansen et al. 2006). Individual RN residual parameter $\sigma_{0j}$ is expressed as a deviation from population average residual parameter $\sigma_{0},$ which together determine the magnitude of variation in observed residuals.
Link functions	$g_{\mu },g_{\sigma },g_{\theta }$	Transformations that facilitate modeling of non‐Gaussian phenotypes and fitness measures on a linear scale.
Fitness	W, $f\left(\theta ,\delta \right)$	A measure of an aspect of an individual's observed survival and reproduction *W*, as predicted by the expectation θ and dispersion δ parameters of distribution *f*. These quantifiable “fitness components” are used to approximate the repeatable, differential rate of zygote production across individuals.
Directional selection	b,β	Selection gradients β quantify the magnitude of direct selection on the population means of reaction norm parameters. Regression coefficients **b** on the scale of a GLMM for fitness can be transformed to estimate these gradients.
Quadratic selection	q,γ	Selection gradients γ quantify the magnitude of direct selection on the (co)variances of reaction norm parameters. Regression coefficients **q** on the scale of a GLMM for fitness can be transformed to estimate these gradients.

RN models are not only useful statistical tools for describing phenotypic variation. Classic theoretical models often assumed that selection acted independently on phenotypes expressed in discrete states of the environment (so‐called *character states*), where the evolution of RN parameters and thus phenotypic plasticity across environments was interpreted as a byproduct of state‐specific selection (Via and Lande 1985; Gomulkiewicz and Kirkpatrick 1992). Many biologists disagreed with this perspective on empirical and theoretical grounds, resulting in historical debates about whether RN parameters should be conceptualized as direct or indirect targets of natural selection (Gavrilets and Scheiner 1993; Scheiner 1993a; Via et al. 1995; Nicoglou 2015). Fortunately, this disagreement is now largely resolved (Futuyma 2021), with evolutionary quantitative genetic theory demonstrating the mathematical equivalence and thus conceptual complementarity of models emphasizing selection on expressed character states or the RNs producing them (de Jong 1995; Martin, Westneat, Wilson, et al. 2025). As such, many contemporary evolutionary frameworks emphasize RN parameters (intercepts, slopes, and residuals) and their underlying mechanisms as putative targets of selection, leading to differential patterns of adaptation and extinction in changing environments (Schlichting and Pigliucci 1998; Ghalambor et al. 2007; Fox et al. 2019). For instance, evolutionary ecologists have long investigated the unique role of both cue‐induced and stochastic phenotypic plasticity in the colonization of novel habitats (Caño et al. 2008; Volis et al. 2015; Hendry 2016; Wang and Althoff 2019). Note that we use the statistical term “stochastic” throughout the text to refer to individual trait variability that is effectively described by a random probability distribution, without necessarily implying that the underlying causal processes are truly random (see Among‐individual differences in RN residuals subsection below). In addition, evolutionary geneticists have shown how plasticity in social environments can magnify heritable variation in mean trait values, accelerating or inhibiting phenotypic evolution in comparison with unresponsive phenotypes (Moore et al. 1997; Bijma and Wade 2008; McGlothlin et al. 2010; Kazancıoğlu et al. 2012). Game theorists and behavioral ecologists have further emphasized the importance of understanding selection on RNs due to the prevalence of fluctuating density‐ and frequency‐dependent selection in social environments (Araya‐Ajoy et al. 2020; McNamara and Leimar 2020; Martin et al. 2023), as well as the role of dynamic environments more generally in selecting for learning mechanisms and emotional states rather than specific behaviors per se (Skinner 1966; Henrich and McElreath 2003; McNamara and Houston 2009; Fawcett et al. 2013; Nakahashi and Ohtsuki 2015; Wright et al. 2022). Distinct genetic control of phenotypic stability and change has also been experimentally demonstrated for diverse phenomena from cold tolerance (Ørsted et al. 2018) to body size (Scheiner and Lyman 1991) and various forms of developmental polyphenism (Suzuki and Nijhout 2006; Projecto‐Garcia et al. 2017), suggesting that differential selection on heritable variation in RN intercepts, slopes, and residuals, as well as differential patterns of genetic integration between RN parameters (Wagner et al. 1997; Tonsor et al. 2013), can in turn have distinct consequences for the evolutionary response to selection (de Jong 1995; Martin, Westneat, Wilson, et al. 2025). Accordingly, divergence has been observed in the RNs of many naturally occurring populations, such as differential plasticity in the growth rates of phytoplankton ( *Thalassiosira pseudonana* ; Schaum et al. 2022), ponderosa pine ( *Pinus ponderosa* ; de la Mata et al. 2022) and single‐leaf pinyon ( *Pinus monophylla* ; Vasey et al. 2022) populations in response to temperature fluctuations and microhabitat heterogeneity. Extensive agricultural and ecological research has also demonstrated the importance of considering evolutionary responses in the continuous functions underlying growth curves across the lifespan (Meyer and Kirkpatrick 2005; Kingsolver et al. 2014; Gomulkiewicz et al. 2018). Despite this strong theoretical emphasis and empirical basis, robust statistical methods have not yet been developed for detecting complex patterns of nonlinear selection on the RNs of labile traits.

Many of the phenotypes commonly studied by evolutionary ecologists are highly labile (i.e., exhibit high degrees of reversible plasticity; Scheiner 1993b) in response to the local environment. This means that repeatable individual differences in the RN underlying these traits tend to account for only a modest proportion of the total variation observed in measurements across space and time (Bell et al. 2009; Fanson and Biro 2015; Cauchoix et al. 2018). This is expected, given that labile traits are often adapted to facilitate flexible responses toward fitness‐relevant variation in the environment (Scheiner 1993b), such as by upregulating circulating testosterone in response to social challenges (Wingfield et al. 1990; Eisenegger et al. 2011), temporarily inducing a fear state in response to odor cues of predation (Mathuru et al. 2012), or regulating alloparental care in response to the quality of the local environment (Guindre‐Parker and Rubenstein 2018; Martin et al. 2020). Conversely, labile traits may also be prone to maladaptive plasticity in response to novel or extreme environmental stressors (e.g., Ghalambor et al. 2015). As such, single measures of labile phenotypes tend to reflect within‐ rather than among‐individual variation, potentially biasing empirical estimates of trait (co)variances and selection gradients estimated across heterogeneous environments (Brommer 2013; Dingemanse and Dochtermann 2013; Niemelä and Dingemanse 2018; Royauté et al. 2018), leading to inaccurate inferences about adaptive evolution (Dingemanse et al. 2021; Martin and Jaeggi 2022). Classical approaches such as the Lande and Arnold (1983) regression framework do not partition repeatable and nonrepeatable differences across phenotypic measurements and, as a consequence, may lead to downwardly biased estimates of selection gradients for labile traits in field research (Dingemanse et al. 2021). Classical methods can also be biased by unmeasured, within‐individual environmental effects on fitness and phenotype that generate spurious signals of selection (Scheiner et al. 2002; Stinchcombe et al. 2002). Using these methods to estimate selection on labile traits with single measures, averages of raw data, or point estimates in multistage analyses can, therefore, increase the risk of biased evolutionary inference (Hadfield et al. 2010), particularly when attempting to understand the adaptation of RNs underlying observed phenotypes across heterogeneous environments.

Fortunately, generalized linear mixed‐effects models (GLMMs) provide a flexible toolkit for estimating RNs from empirical data, as well as for modeling the effects of RNs on fitness and other biological outcomes of interest. In biology, GLMMs with random slopes are often referred to as “random regression” models (Henderson 1982), due to the specification of individual variation in multiple parameters of the regression line, while GLMMs with random residual components are referred to as “double hierarchical” models (Rönnegård et al. 2010). We rely herein on the more general GLMM terminology that integrates and encompasses both models as special cases. Current variance‐partitioning methods rely on the use of multiresponse/multivariate GLMMs with covarying random effects to model selection, which effectively account for uncertainty in individuals' RNs and their estimated effects (Hadfield et al. 2010). This approach has been repeatedly introduced to selection studies of RNs in a variety of contexts, demonstrating its broad applicability (e.g., Brommer et al. 2012; Houslay and Wilson 2017; Arnold et al. 2019), and can be further extended to provide a veritable treasure chest of biological insights (Blows 2007). For example, such models can be used to identify trajectories of phenotypic conservation and divergence among closely related populations (Royauté et al. 2020), discover latent behavioral characters among multiple traits (Araya‐Ajoy and Dingemanse 2014; Martin et al. 2019), or calculate genetic responses to directional selection (Stinchcombe et al. 2014). Therefore, multiresponse GLMMs with covarying random effects can be used to accomplish many empirical goals with relative ease, while also avoiding statistical bias due to uncertainty in RNs. Within the broader study of function‐valued and so‐called “infinite‐dimensional” traits (Kirkpatrick and Heckman 1989), of which the highly labile phenotypes considered here are a subset, a variety of GLMMs integrating generalized additive functions such as splines have also developed, which can be used to estimate continuous variation in directional selection on highly nonlinear RNs across environments (Stinchcombe et al. 2012; Gomulkiewicz et al. 2018).

Despite their benefits, these commonly used GLMMs cannot detect nonlinear selection on RNs (i.e., disruptive, stabilizing, and correlational selection) because the random effect covariance is defined as an average measure of linear dependency among fitness and phenotype. By failing to describe the curvature of the adaptive landscape, and thus the ecological phenomena generating fitness saddles, ridges, domes, and cliffs (Lande and Arnold 1983; Blows and Brooks 2003; Blows 2007; Vercken et al. 2012), random effect covariance models can provide an incomplete and potentially misleading perspective on the biological processes driving and constraining multivariate evolution. In nonrandomized experiments or field settings, ignoring nonlinear selection can further generate biased estimates of directional selection gradients, in addition to biased predictions of the evolutionary response to selection on the expectations and (co)variances of RN parameters (Arnold et al. 2001; Morrissey et al. 2012; Pick et al. 2022). Therefore, despite their clear utility, current random effect covariance models can also limit robust tests of adaptive theory, which often predicts that stabilizing, disruptive, and/or correlational selection will shape RN evolution (e.g., Wagner et al. 1997; Gavrilets and Hastings 1994). This inhibits accurate predictions of phenotypic evolution more generally (Bulmer 1971; Lande and Arnold 1983; Arnold et al. 2001; de Villemereuil et al. 2020).

Here we address this challenge by introducing multiresponse/multivariate GLMMs for unbiased estimation of nonlinear selection on RNs, building on well‐established approaches to estimating linear selection (e.g., Brommer et al. 2012; Houslay and Wilson 2017; Arnold et al. 2019; Araya‐Ajoy et al. 2023). The proposed GLMMs are applicable to any labile and repeatedly measured phenotype. We begin by reviewing GLMMs for estimating RNs from longitudinal, repeated measures data (Westneat et al. 2013; O'Dea et al. 2021) and formally introduce multiresponse/multivariate models estimating linear and nonlinear selection on these RNs, applicable to both Gaussian and non‐Gaussian measurements. We then consider their implementation in a Bayesian framework, using simulations to validate that the proposed models are unbiased for statistical inference. We also explore statistical power for Bayesian hypothesis tests across a range of sampling designs and selection effect sizes. A step‐by‐step guide is then laid out for when and how the model should be applied, and a guided tutorial is further provided (see Data Availability Statement) to assist researchers in implementing and interpreting these models for their own data using the Stan probabilistic programming language (Carpenter et al. 2017). Our principal goals in the present study are, therefore, to conceptually and formally introduce this novel method, to explain its interpretation and implementation, and to statistically validate its application for empirical research, while also providing a workflow and code to aid in this process.

## 2 Modeling Nonlinear Selection on RNs

The models we propose in this section are straightforward extensions of the multiresponse/multivariate random effects GLMMs discussed above. Thus, much of the ground covered here is based on an extensive and longstanding body of work for the measure of selection on complex traits. The novelty of our trait‐based approach for the study of selection on RNs is that it shifts the estimation of fitness effects from random effect covariances to flexibly parameterized linear and nonlinear selection coefficients. This approach builds on a long tradition of measurement error models in biostatistics (Loken and Gelman 2017; Ponzi et al. 2018; Martin and Jaeggi 2022), also known as structural equation (Bollen and Noble 2011; Araya‐Ajoy and Dingemanse 2014; Martin et al. 2019) or errors‐in‐variables models (Dingemanse et al. 2021), which allow for latent trait values such as RN intercept, slope, and residual parameters to simultaneously affect multiple response models. The basic structure of these models has been previously introduced in the broader context of phenotypic selection analysis by Ponzi et al. (2018), Dingemanse et al. (2021), and Araya‐Ajoy et al. (2023), who considered models of selection on repeatable trait values. Here, we generalize and extend these models to allow for estimating nonlinear selection on RN intercepts, slopes, and residuals (and any other distributional parameters of interest), as well as to estimate directional and quadratic selection gradients on RN parameters with non‐Gaussian phenotype and fitness measures.

### 2.1 Reaction Norm Model

The first step in any selection analysis is to define the trait of interest. For repeatedly expressed traits that exhibit plasticity, the “traits” of interest may be latent properties of a RN, which researchers can estimate as functional parameters. As shown in Figure 1, individual variation in a linear RN can be decomposed into underlying repeatable differences in individuals' RN intercept $\mu_{0}$, slope $\beta_{x}$, and residual parameters $\sigma_{0}$. Note that we use $\beta_{x}$ here to reference any slope defined over a nonsocial environmental state (see Martin and Jaeggi 2022 for a treatment of social effects). Table 1 provides a glossary of formal notation and terminology used throughout the paper. GLMMs effectively describe the RNs of Gaussian or non‐Gaussian phenotypes using additive linear functions on a transformed latent scale (Bolker et al. 2009; de Villemereuil et al. 2016), which may include highly flexible and nonparametric basis functions such as splines for describing complex patterns of nonlinearity in trait expression (Stinchcombe et al. 2012; Gomulkiewicz et al. 2018). Extensive prior work has been done on appropriate study design and GLMM implementation for RN research in evolutionary ecology (e.g., see Nussey et al. 2007; Martin et al. 2011; Dingemanse and Dochtermann 2013; O'Dea et al. 2021 among others). Therefore, we avoid reviewing this material in detail here, instead focusing on the introduction of a general form and notation for RN models of any labile trait.

Consider a GLMM for repeated measure *t* of individual *j*, who expressed labile phenotype $z_{\mathrm{jt}}$ in environmental state $x_{\mathrm{jt}}$. The distribution of measurements can be predicted using a probability function $f\left(\mu ,\sigma \right)$ with mean, location, or rate parameter μ and dispersion, shape, or scale parameter σ (e.g., as with normal, gamma, and beta distributions). Link functions $g_{\mu }$ and $g_{\sigma }$ are used for modeling the vectors μ and σ across observations so that the RN parameters $\mu_{0}$, $\beta_{x}$, and $\sigma_{0}$ can be expressed as additive linear effects on a transformed scale, irrespective of the assumed distribution of the raw data. For instance, $g_{\mu }=\text{identity}\left(\mu \right)$ and $g_{\sigma }=\log\left(\sigma^{2}\right)$ are sensible choices for a Gaussian measure. The generalized form of the model is given by

(1) $$z_{\mathrm{jt}}∼f\left(\mu_{\mathrm{jt}},\sigma_{j}\right)$$

$$g_{\mu }\left(\mu_{\mathrm{jt}}\right)=\mu_{0}+\mu_{0j}+\left(\beta_{x}+\beta_{\mathrm{xj}}\right)x_{\mathrm{jt}}$$

$$g_{\sigma }\left(\sigma_{j}\right)=\sigma_{0}+{\sigma_{0}}_{j}$$

$$\begin{array}{c} {\left[\mu_{0}^{⊤},\beta_{x}^{⊤},\sigma_{0}^{⊤}\right]}^{⊤}∼\mathrm{MVN}\left(0,P\right):\;P=\left[\begin{array}{ccc} \mathrm{var}\left(\mu_{0}\right) & \ldots & \ldots \\ \mathrm{cov}\left(\beta_{x},\mu_{0}\right) & \mathrm{var}\left(\beta_{x}\right) & \vdots \\ \mathrm{cov}\left(\sigma_{0},\mu_{0}\right) & \mathrm{cov}\left(\sigma_{0},\beta_{x}\right) & \mathrm{var}\left(\sigma_{0}\right) \end{array}\right] \end{array}$$

where ⊤ indicates the transpose operator. Here $\mu_{0}$, $\beta_{x}$, $\sigma_{0}$ are the average values for the RN intercept, slope, and residual parameters in the population, expressed on the scale of the link functions. Repeatable individual differences in RN parameters are in turn estimated as deviations from these averages using random effects $\mu_{0j}$, $\beta_{\mathrm{xj}}$, and $\sigma_{0j}$. Therefore, each observation $z_{\mathrm{jt}}$ is predicted by an individual‐specific function $f\left(\mu_{\mathrm{jt}},\sigma_{j}\right)$, with parameters $\mu_{\mathrm{jt}}$ and $\;\sigma_{j}$ determining the expected location and scale of variation in the individual's distribution of phenotypes expressed within and across environments. An individual with a large $\mu_{\mathrm{jt}}$ and small $\;\sigma_{j}$ will, for example, have a high phenotypic mean across instances of trait expression in the environment characterizing measure *t*, with low phenotypic variability around their mean. If selection acts on these individual parameters, it can change the average location and scale of the phenotypic distribution expected for individuals in a population. For simplicity, the model assumes environmental exposures ***x*** are randomized across individuals, but it may be necessary in nonexperimental contexts to center covariates within individuals for appropriate scaling of RN slopes (van de Pol and Wright 2009; Araya‐Ajoy et al. 2015; Westneat et al. 2020; Fay et al. 2022). The magnitude of among‐individual (co)variance in RN parameters is described by the P matrix. See Equations (S1) and (S2) in the Appendix S1 for details on how to estimate the total repeatable variation in the phenotype explained by individuals' RNs.

### 2.2 Among‐Individual Differences in RN Residuals

The functional role of the RN residual parameters $\sigma_{0}$ merits further discussion, as their interpretation can be ambiguous. These individual effects are modeled on the dispersion σ of the phenotypic distribution, rather than the expectation μ (Equation 1). Phenotypic variance due to dispersion is generally interpreted as noise or measurement error around individuals' repeatable mean trait values (Brommer 2013), which are determined by the expression of RN intercepts $\mu_{0}$ and slopes $\beta_{x}$ across measured environments. However, the residuals of labile traits may also contain repeatable and fitness‐relevant variation in how organisms intrinsically regulate their phenotype (Westneat et al. 2015), such as in their assessment and response toward developmental noise within a given environment (Gavrilets and Hastings 1994; Hansen et al. 2006; Mitchell et al. 2021). Such repeatable *among*‐individual differences in *within*‐individual variation, described by $\sigma_{0}$, may arise from a variety of mechanisms regulating patterns of stochastic expression in behavior or other labile traits (Prentice et al. 2020). For instance, stochasticity can be generated through the repeatable activities of the organism, such as by random sampling of the environment, which can be shaped via reinforcement and punishment to facilitate adaptive learning in novel or uncertain environments (Niv et al. 2002; Barrett 2011; Wright et al. 2022). Consequently, intrinsic variability may evolve in conjunction with learning mechanisms to track unpredictable shifts in fitness optima during development (Borenstein et al. 2008). Predation may also select for greater variability in movement, reducing predators' capacity to predict prey escape trajectories (Hugie 2003; Moore et al. 2017). Alternatively, reduced variability may instead be adaptive for reputation formation and trust in repeated social interactions (McNamara and Leimar 2010). In either case, selection would be expected to occur on $\sigma_{0}$. Stochasticity may also result from exogenous factors, such that individual differences in $\sigma_{0}$ reflect how organisms regulate in response to the environment. For example, when environmental states fluctuate rapidly in an unpredictable and uncontrollable manner, negative selection may act on the RN residual parameter to promote phenotypic canalization, decreasing susceptibility of the phenotype to developmental perturbation (Flatt 2005; Siegal and Leu 2014; Westneat et al. 2015). Alternatively, when factors such as resource availability change stochastically, selection may instead favor wider phenotypic distributions, as organisms benefit from responding to and taking advantage of the local affordances provided by these fluctuations. For instance, using the modeling framework proposed here, Martin, Westneat, Nakagawa, et al. (2025) have recently demonstrated that selection for offspring quality favors larger residual clutch size variation among house sparrows ( *Passer domesticus* ). This suggests that females who are more responsive to stochastic environmental fluctuations are better able to maintain the mass of their offspring across breeding attempts. Alternatively, selection for offspring quantity favors females with tighter distributions, whose more canalized reproductive behavior leads to consistently higher clutch sizes across breeding attempts independently of local conditions.

In empirical research, it will often be challenging to distinguish variance in residuals due to intrinsically stochastic variability or unmeasured processes of cue‐induced plasticity and individual‐by‐environment interaction (Westneat et al. 2015; Prentice et al. 2020). Estimates of $\mathrm{var}\left(\sigma_{0}\right)$ in the field may, for example, reflect repeatable functional interactions between unmodelled RN slopes and stochastic environmental exposures. Therefore, caution is warranted when inferring the mechanistic underpinnings of $\sigma_{0}$ outside of well‐controlled experiments. Poorly specified statistical models, in which predicted residual processes do not accurately describe observed phenotypic variance, will also inhibit accurate biological inference of RNs (Mitchell et al. 2020; Ramakers et al. 2020). Nonetheless, to the degree that individual differences in residuals are repeatable across time and not due to unbalanced sampling or pseudo‐repeatability (Dingemanse and Dochtermann 2013), selection can still shape RN residuals, irrespective of whether within‐individual deviations arise from mechanisms of intrinsically stochastic or cue‐induced trait expression. Therefore, we suggest that researchers in both observational and experimental systems focus their attention on functionally interpreting and operationally defining RN residual parameters with respect to theoretically motivated RN slopes, defined over measured dimensions of environmental change (Figure 1).

### 2.3 (Non)Linear Selection Model

With the RN model (Equation 1) in place, selection can now be estimated on the individual‐specific parameters ${\mu_{0}}_{j}$, $\beta_{\mathrm{xj}}$, and ${\sigma_{0}}_{j}$ by expanding the GLMM to include an additional response model predicting measure *t* of fitness component or proxy ***W***. Linear ***b*** and quadratic ***q*** selection coefficients, as well as other more complex forms of nonlinear selection, can then be estimated directly for the RN parameters.

(2) $$\begin{array}{c} z_{\mathrm{jt}}∼f\left(\mu_{\mathrm{jt}},\sigma_{j}\right) \end{array}$$

$$g_{\mu }\left(\mu_{\mathrm{jt}}\right)=\mu_{0}+\mu_{0j}+\left(\beta_{x}+\beta_{\mathrm{xj}}\right)x_{jt}$$

$$g_{\sigma }\left(\sigma_{j}\right)=\sigma_{0}+{\sigma_{0}}_{j}$$

$${\left[\mu_{0}^{⊤},\beta_{x}^{⊤},\sigma_{0}^{⊤}\right]}^{⊤}∼\mathrm{MVN}\left(0,P\right)$$

$$W_{\mathrm{jt}}∼f\left(\theta_{\mathrm{jt}},\delta \right)$$

$$\begin{array}{cc} g_{\theta }\left(\theta_{\mathrm{jt}}\right)= & W_{0}+W_{0j}+b_{1}\mu_{0j}+b_{2}\beta_{\mathrm{xj}}\\ & +b_{3}\sigma_{0j}+q_{1}\mu_{0j}^{2}+q_{2}\beta_{\mathrm{xj}}^{2}+q_{3}\sigma_{0j}^{2}\\ & +q_{4}\mu_{0j}\beta_{\mathrm{xj}}+q_{5}\mu_{0j}\sigma_{0j}+q_{6}\beta_{\mathrm{xj}}\sigma_{0j} \end{array}$$

$$W_{0}\sim N\left(0,\mathrm{sd}\left(W_{0}\right)\right)$$

**FIGURE 2:**
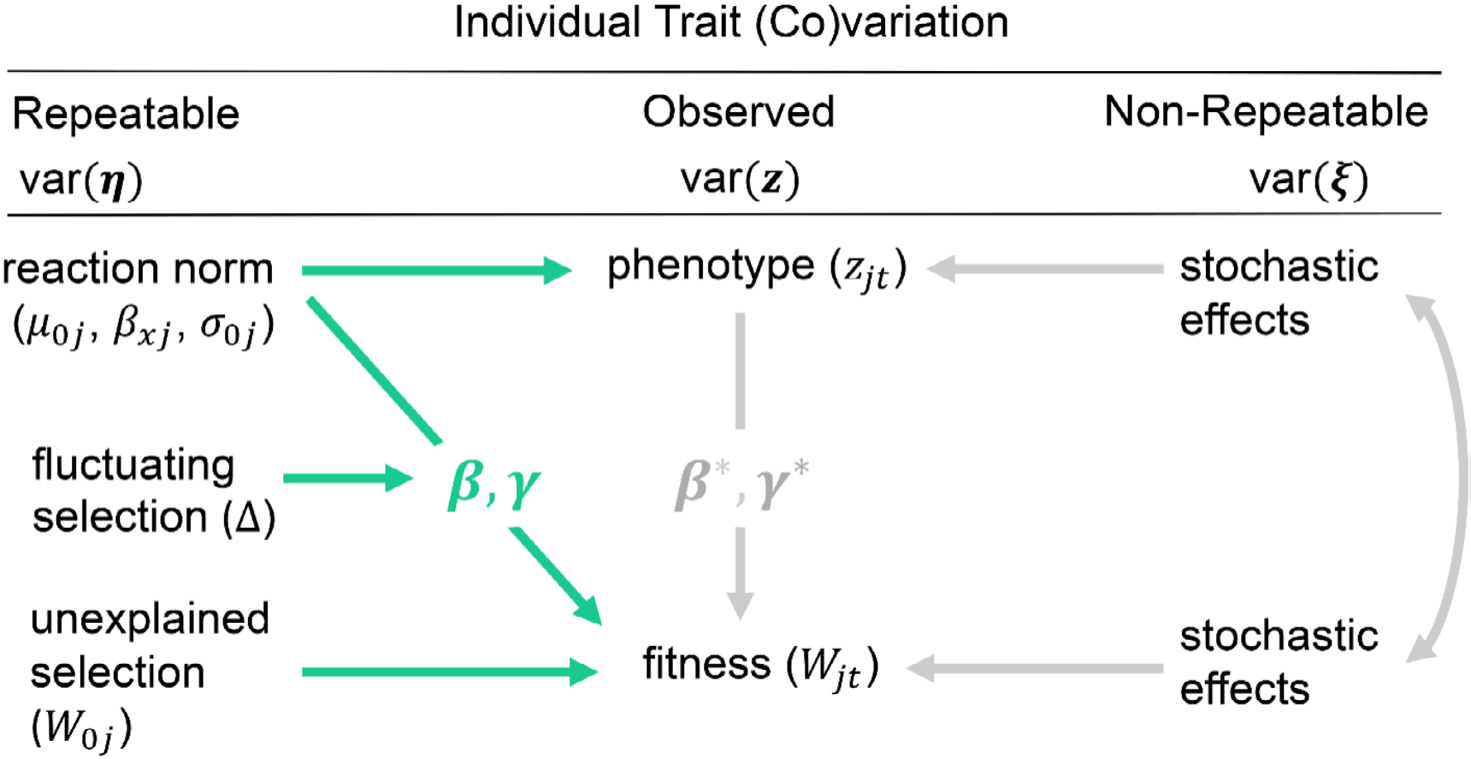
Removing nonrepeatable effects from selection gradients. The diagram shows causal pathways (directional arrows) by which repeatable (green) and nonrepeatable (gray) effects can influence selection gradients of fitness (W) on phenotype (z). Nonrepeatable, stochastic effects influence both fitness and phenotype (directional arrows) and may be correlated (double‐headed arrow), introducing statistical noise into the selection analysis. This leads to biased directional $\beta^{*}$ and quadratic gradients $\gamma^{*}$ when observed variance in the phenotype var(***z***) is used to estimate selection across environments. However, if the (non)linear relationships between phenotype and fitness are modeled independently of stochastic effects on the phenotype var(ξ), using RN parameters $\mu_{0},\beta_{x}$, and $\sigma_{0}$ (Equations 1 and 2), unbiased selection gradients β and γ can be estimated (Equations S3–S5: Appendix S1) directly for repeatable among‐individual differences in the phenotype var(η) (Equations S1 and S2: Appendix S1). Spatiotemporal fluctuations Δ can also occur in these selection gradients, which can be described by interactive selection effects (see Equations S8 and S9.2: Appendix S1 for further discussion), and any repeatable among‐individual differences in fitness unexplained by RN parameters can be estimated with random effects $W_{0}$ when repeated fitness measures are available (Equation 2).

Fitness component *W* for individual *j* at measurement *t* is described by a GLMM with expectation parameter θ and dispersion parameter δ. Therefore, the full multivariate GLMM simultaneously estimates the RN parameters and their accompanying linear and quadratic selection effects in the fitness model. Figure 2 visualizes this model structure and explains how it avoids bias by partitioning repeatable sources of (non)linear association between phenotype and fitness. Parameter $W_{0}$ is the average fitness on the transformed scale given by link function $g_{\theta }$. When repeated measures *t* are available for a given fitness component, an individual random effect $W_{0j}$ should be estimated to capture repeatable among‐individual differences in fitness that are not due to the modeled phenotypes (i.e., unexplained selection). If only a single fitness measure is available, $\mathrm{sd}\left(W_{0}\right)$ cannot be identified separately from fitness residual dispersion δ, so these individual‐level random effects should instead be excluded from the analysis.

The polynomial regression in Equation (2) can be used to infer short‐term population trajectories on the adaptive landscape, under the assumption that a quadratic function effectively approximates the local shape of the individual selection surface on the latent transformed scale (Lande and Arnold 1983; Phillips and Arnold 1989). However, the values of the ***b*** and ***q*** regression coefficients should only be interpreted as measures of directional β and quadratic γ selection gradients when fitness is a mean‐scaled Gaussian response, after appropriately scaling the coefficients (Stinchcombe et al. 2008; Dingemanse et al. 2021). Analytic expressions can also be used for direct interpretation of coefficients in a log‐normal fitness model (Bollen et al. 2019; Morrissey and Goudie 2022). However, in the general case, it will be necessary to further process these regression coefficients from the fitness model to infer directional and quadratic selection on the scale of the original data. This process is detailed further in the Equation (S3): Appendix S1 and accompanying coding tutorial (Data Availability Statement), along with consideration of standardizing selection gradients for ease of comparison across link functions and statistical distributions (Equations S4 and S5: Appendix S1).

The model structure presented above is intentionally simplified to aid comprehension and clarity, focusing attention on selection of a simple linear RN for a single trait. This, of course, ignores many common considerations relevant for any regression analysis, such as the inclusion of additional adjusted effects, more complex nonlinear selection functions such as splines (Equation S6: Appendix S1), or other individual‐level parameters (Equation S7: Appendix S1). More detailed consideration on these matters is provided in the Appendix S1, along with discussion of when fluctuating selection on RNs (Δβ and Δγ; Figure 2) is expected to occur and how it can be estimated (Equation S8: Appendix S1). Therefore, it is important to emphasize that the simplified structure in Equation (2) is not a limitation of the proposed framework. Using the same general strategy outlined here for the single trait case, as well as the broad toolkit available for any multivariate regression, the (non)linear selection model can be straightforwardly extended to RNs with many additional terms, as well as to multiple potential phenotypes and fitness components (see Equation S9: Appendix S1 for a more generalized formulation). The key innovation proposed here is simultaneously estimating random individual RN parameters and their nonlinear selection effects on fitness in a single multivariate GLMM. This can in principle be accomplished for model structures of any desired complexity.

## 3 Statistical Implementation

### 3.1 Bayesian Estimation

The proposed (non)linear selection model cannot currently be estimated using popular GLMM software packages, due to the need for latent RN parameters to be simultaneously estimated with random and fixed effects across different response models. Fortunately, the Stan probabilistic programming language (Carpenter et al. 2017), which relies on cutting‐edge and computationally efficient Markov Chain Monte Carlo (MCMC) sampling algorithms, provides the flexibility needed for estimating these novel GLMMs within a Bayesian framework. Researchers unfamiliar with the general motivations of Bayesian inference are encouraged to see McElreath (2020) and Gelman et al. (2020) for helpful tips on developing an effective workflow for data analysis. We provide a detailed, guided tutorial (see Data Availability Statement) for implementing the models presented here in Stan.

Prior distributions need to be specified for all the population‐level parameters in a Bayesian model. While flat or highly diffuse priors are often recommended in the literature (e.g., Ellison 2004; de Villemereuil et al. 2016; Houslay and Wilson 2017), weakly informative or regularizing priors, which place relatively low probability on extreme effect sizes, facilitate more robust and efficient inferences with limited sample sizes and should generally be preferred over flat priors (Gelman and Tuerlinckx 2000; Lemoine 2019; McElreath 2020). This does not necessarily require strong a priori assumptions, as general‐purpose priors can be used to increase the robustness of parameter estimates even in a state of relative ignorance about the true effect size. For highly complex selection models with many parameters, prior regularization will also be particularly useful for ensuring model identifiability. See Lemoine (2019), McElreath (2020), and Gelman et al. (2020) for more detailed discussion and recommendations.

### 3.2 Model Validation

To provide a general validation for empirical application, we explored whether the proposed model facilitates unbiased Bayesian estimation across a broad range of selection effect sizes and sample size conditions. Sampling conditions were continuously and uniformly varied for the total number of subjects $N\sim U\left(100,1000\right)$, as well as the number of repeated phenotypic measurements $t_{z}\sim U\left(3,7\right)$ and repeated fitness measures $t_{w}\sim U\left(1,5\right)$ per subject. Selection effects ***b*** and ***q*** were simulated so that standardized selection gradients β,$\gamma \sim U\left(0.1,0.5\right)$ ranged from small effects, which are most frequently observed in natural populations (Hendry 2020; Arnold 2023), to large effects. For simplicity, repeatable among‐individual differences in the RN parameters were fixed to $\mathrm{sd}\left(\left[\mu_{0},\beta ,\sigma_{0}\right]\right)=1$ to facilitate standardized interpretation of selection gradient effect sizes. Correlations among RN parameters were drawn from $\mathrm{cor}\left(\left[\mu_{0},\beta ,\sigma_{0}\right]\right)\sim \mathrm{LKJ}\left(5\right)$ such that marginal correlations were weakly centered near zero with low probability beyond ~∣0.5∣. The residual standard deviation of the phenotype was fixed to $\text{sqrt}\left(\exp\left(\sigma_{0}\right)\right)=1.41$, so that repeatable and residual random effect variances were 1 and 2, respectively. This resulted in each RN parameter exhibiting modest repeatability, $R=0.2=\frac{1}{3*1+2}$ in the absence of phenotypic correlations. Unexplained selection was also fixed to $\mathrm{sd}\left(W_{0}\right)=1$ for the fitness model. We simulated 500 datasets for analysis, with each dataset representing a randomly selected combination of sampling and selection effect sizes. Phenotype and fitness were assumed to be Gaussian for computational efficiency.

Bias was calculated by the difference between the simulated (true) selection effect size and the estimated posterior median effect size, such that expected values centered on 0 provided support for unbiased inference from the model. The root mean squared deviation (RMSD), calculated across each posterior distribution as the square root of the average squared difference between the true and estimated selection effect, was used to quantify overall accuracy incorporating uncertainty around the expected effect size. Classical frequentist methods define power with respect to a binary decision rule based on the desired significance level of a null hypothesis test. In Bayesian analysis, power is not precisely defined but may instead refer to the continuous level of support provided for a direct (rather than null) hypothesis test, such as the posterior probability of positive selection occurring on a trait ($p_{+}$). The power of a Bayesian analysis thus reflects how confident a model is likely to be in the existence and direction of a true selection effect, with $p_{+}$ = 0.5 indicating no confidence (+ and − values are equally likely) and $p_{+}$ = 1.0 indicating complete confidence in the effect.

**FIGURE 3:**
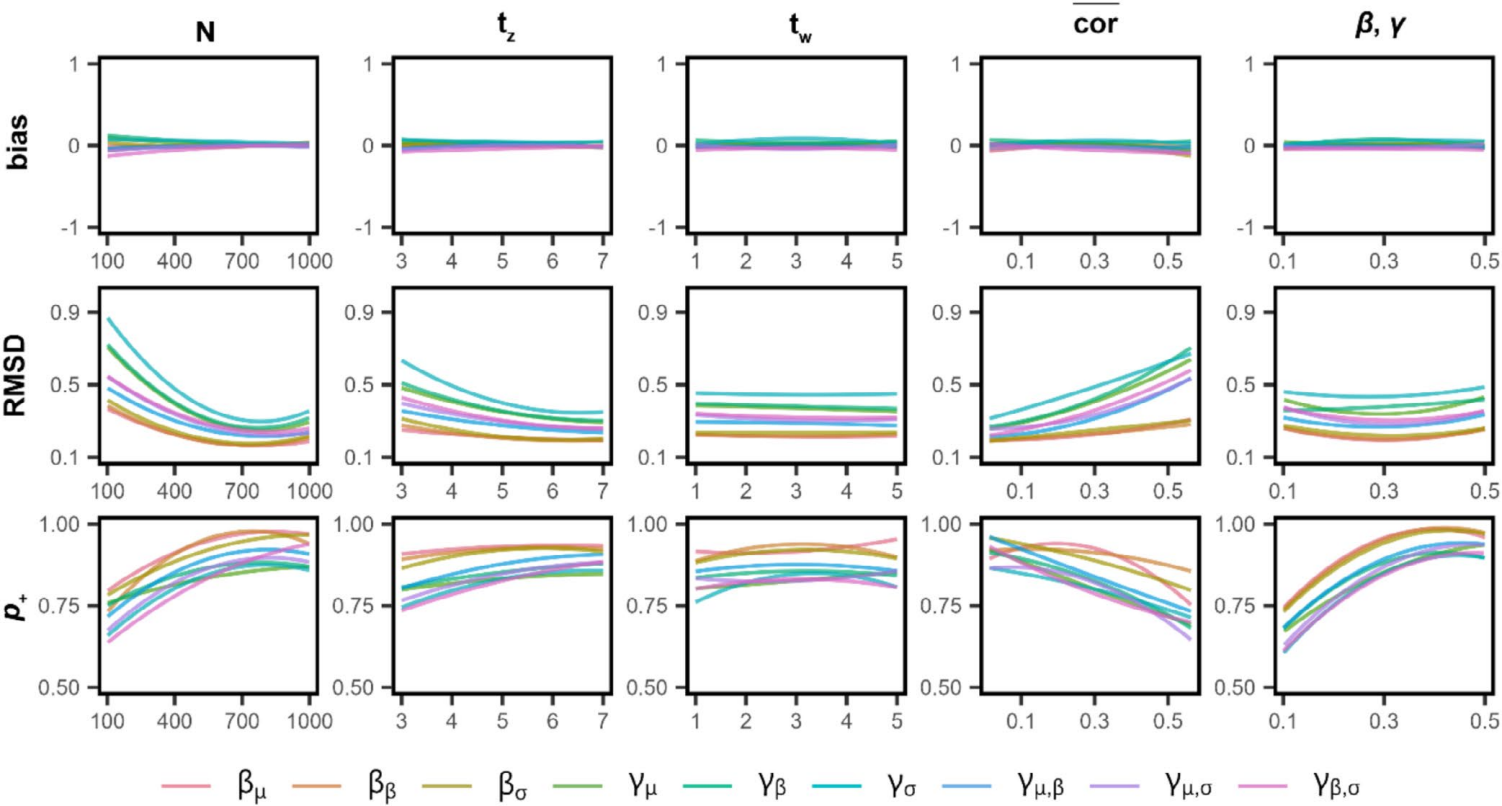
Model performance for inferring (non)linear selection on RNs. Results are shown for inferred selection gradients across 500 simulated datasets used to estimate the (non)linear selection model for RNs (Equation 2) with Gaussian phenotype and fitness measures. Each row shows a model performance metric: Bias (true value—median estimate), root mean square deviation (RMSD; the square root of the average squared deviation between true and estimated posterior values), and the posterior probability (“power”) in support of positive directional or quadratic selection ($p_{+}$). Columns show how these metrics change across simulated datasets as a function of the number of subjects (*N*), the number of phenotypic measures per subject (*t*_z_), the number of fitness measures per subject (*t*_w_), the mean absolute correlation among RN parameters ($\bar{\mathrm{cor}}$), and the size of directional (β) and quadratic (γ) selection effects. General patterns were summarized using second‐order polynomials across conditions, which are color‐coded by RN parameter (bottom legend).

Results across simulated datasets are visualized in Figure 3, with second‐order polynomial lines plotted across datasets to infer general patterns expected in empirical research. Across simulated conditions, expected bias in estimated selection gradients remained centered on 0, indicating that the proposed model provides unbiased inferences across effect and sample sizes. As expected, we find that RMSD decreases and $p_{+}$ increases with a larger number of subjects (N) and repeated measures of the phenotype ($t_{z}$), though total N had a relatively stronger impact than $t_{z}.$ Selection effect sizes (β,γ) had little impact on RMSD but greatly affected $p_{+}$ in a manner comparable to N. Larger absolute phenotypic correlations among RN parameters ($\bar{\mathrm{cor}}$) reduced both RMSD and $p_{+}$, particularly for quadratic selection, with much less influence on directional selection. In contrast, repeated fitness measures ($t_{w}$) had little impact on either RMSD or $p_{+}$, suggesting that a single measure of fitness is generally sufficient for inferring selection.

Overall, total sample size, selection effect sizes, collinearity among RN parameters, and the number of repeated phenotypic measures together determined the accuracy of and power to detect selection gradients. Both accuracy and power were consistently lower for quadratic selection than for directional selection across sample and effect sizes, with stabilizing/disruptive selection gradients exhibiting the highest RMSD across conditions. This implies that research particularly focused on detecting quadratic selection of RNs will require relatively larger samples to attain confident inferences. As with any multivariate selection model, these results also show that larger sample sizes and sufficient repeated measurements promote more robust hypothesis testing, particularly in the presence of weak selection. As a rule of thumb, sample sizes of approximately *N* = 500+ subjects, where the effect of total sample size on model performance begins to diminish, will be particularly ideal for attaining informative estimates, though repeated phenotypic measures can partially compensate for the loss of information in smaller samples. The effects of RN parameter correlations on RMSD and power also show that nonlinear selection in particular will be easier to detect when RN parameters vary quasi‐independently among individuals within a population.

### 3.3 Workflow for Application

Having introduced and validated the proposed modeling framework, we now briefly sketch a typical four‐step workflow for applying these models in R. See Martin, Westneat, Nakagawa, et al. (2025) as well for a recent worked empirical example applying the proposed framework to (non)linear selection on clutch size RNs via multiple fitness components.

#### 3.3.1 Step 1. Consider the Study Design

Before any coding is done, researchers should consider whether the proposed model is well‐suited to their study design. Some key questions are whether there are theoretical, empirical, and/or statistical motivations for hypothesizing that RN parameters are under differential selection; whether the study design facilitates repeated measurements of the same individuals over time, or analogously for genotypes or lineages, clonal organisms or kin exposed to different environments in a breeding experiment; and whether it is optimal, given biological considerations and pragmatic constraints, to prioritize gathering a larger sample size and/or more repeated measures of phenotypes on the same individuals (Figure 3).

#### 3.3.2 Step 2. Decide on the Model Structure

The modeling framework can extend far beyond the simple case presented in the main text (Equations 1 and 2), using all the standard tools available for GLMMs. Some key questions are how many traits and fitness components are relevant for the study goals (see Equation S9: Appendix S1 for multivariate extensions); how many environmental factors are pertinent for estimating RN slopes; what selection gradients are of principal interest; what statistical distributions are appropriate for predicting measurements; and what additional fixed and/or random effects should be adjusted for to ensure meaningful interpretation of among‐individual variation in fitness and phenotype (see Appendix S1 for further discussion).

#### 3.3.3 Step 3. Code and Estimate the Model

A detailed tutorial is provided in the accompanying repository (Data Availability Statement) demonstrating how to code the proposed models in Stan using simulated data in R. Standard considerations for regression analysis should be applied at this stage to decide on the final structure for biological inference. It may, for instance, be pertinent to use model selection, averaging, or related approaches to choose between nested structures of varying complexity (see Gelman et al. 2020 for further guidance).

#### 3.3.4 Step 4. Calculate Selection Gradients

As emphasized above, the **b** and **q** coefficients in Equations (2) and (S9.1): Appendix S1 (as well as any accompanying fluctuating selection terms, Equations (S8) and (S9.2): Appendix S1) should not be directly interpreted as directional and quadratic selection gradients. A general solution irrespective of model structure is to code the estimated fitness function in R and numerically derive posterior distributions for selection gradients (Equation S3: Appendix S1). This is relatively straightforward because MCMC samples of the model parameters can be iteratively analyzed to generate posterior samples of selection gradients (see tutorial for details). Selection gradients can then be standardized for ease of comparison (Equations S4 and S5: Appendix S1), as well as summarized and visualized using standard Bayesian approaches (see tutorial and Gelman et al. 2020; McElreath 2020 for general guidance).

## 4 Conclusion

Studying selection on highly labile traits is essential for explaining how and why organisms adapt to environmental change. RN models are a crucial tool for characterizing such phenotypes, but their application to selection analysis remains hampered by the limitations of current methods. A major challenge is to avoid inferential bias caused by nonrepeatable, stochastic effects and other sources of measurement error in RNs and their fitness effects (Hadfield et al. 2010; Figures 1 and 2). A common solution is to use multiresponse/multivariate random effect GLMMs to account for uncertainty in RNs while estimating the covariance between fitness and RN parameters. However, this approach restricts analyses to focus on linear effects and directional selection. Ignoring quadratic selection caused by nonlinear effects fundamentally inhibits researchers' capacity to study the adaptive landscape of labile traits (Bulmer 1971; Arnold et al. 2001; Blows and Brooks 2003). To overcome this limitation, we proposed a novel Bayesian GLMM framework for studying complex patterns of nonlinear selection on RNs, which we validated over a broad range of possible effect sizes and sampling conditions (Figure 3). Simulations showed that the model facilitates unbiased estimates of selection, with desirable accuracy and statistical power under reasonable sampling conditions for many long‐term field projects. This framework synthesizes the well‐established Lande and Arnold (1983) approach to error‐free selection analysis with latent variable (also known as measurement error or error‐in‐variables) models (Ponzi et al. 2018; Dingemanse et al. 2021; Martin and Jaeggi 2022) and random regression, double hierarchical, multiresponse GLMMs (Brommer et al. 2012; Westneat et al. 2013; Houslay and Wilson 2017; Arnold et al. 2019; O'Dea et al. 2021). These models can be applied to estimate directional and quadratic selection irrespective of the distribution of the data and the potential nonlinearity of the RN or fitness function, allowing researchers to construct more realistic models of the processes underlying their measurements. This focuses attention on accurate description of observed data rather than the restrictive assumptions of linear regression. With the analytic toolkit of evolutionary quantitative genetics (Walsh and Lynch 2018; Arnold 2023), estimates from these models can also be transformed to quantify selection gradients, visualize multivariate selection, and predict the ongoing adaptation of labile traits in the face of contemporary environmental change.

Notwithstanding these benefits, it is also important for empiricists to keep in mind the limitations of the proposed framework. Moderately large sample sizes will often be necessary for quantifying selection on RN parameters with a high degree of accuracy and statistical power, particularly slopes and residual scales as well as quadratic selection gradients (Figure 3). The challenge of achieving sufficient statistical power will, of course, only be more severe for models containing many RN components, phenotypes, and/or fitness components (Equation S9: Appendix S1). This suggests that further developing the proposed models to incorporate dimension–reduction techniques will be a valuable direction for future research, building on established methods in the broader study of function‐valued traits (Meyer and Kirkpatrick 2005; Kingsolver et al. 2014) and multivariate selection (Blows and Brooks 2003). More generally, empiricists should think carefully about the appropriateness of their system and dataset when endeavoring to investigate nonlinear selection on RNs. Modest sample sizes, particularly for high‐dimensional RNs, are unlikely to bring clear biological insights, given that selection gradients in nature tend to be small in size (Hendry 2020; Arnold 2023).

Using the proposed models also requires greater investment in learning probabilistic programming and Bayesian inference than is typical for classical applications of Lande and Arnold (1983) selection analysis. This can increase the risk of inferential error in the process, as well as the time and computational investment required. However, these costs are outweighed by the benefits achieved through a rigorous analysis of nonlinear selection, avoiding the pitfalls of estimating selection with error‐prone measurements of labile traits (Dingemanse et al. 2021). To this end, we have provided a detailed tutorial to aid empiricists in coding their own models in Stan (Data Availability Statement). It is important to keep in mind that GLMMs in general are sophisticated models that remain challenging to master but are, nonetheless, essential tools for robust inference in many areas of evolutionary ecology (e.g., Bolker et al. 2009; Wilson et al. 2010; Dingemanse and Dochtermann 2013). We believe that the growing use of Stan reflects a need for standard GLMMs to be taken even further for accomplishing the contemporary inferential challenges facing our field (e.g., Ranjeva et al. 2019; Acker et al. 2023; Bliard et al. 2024).

It may not be well‐motivated to conceptualize RN parameters as causal targets of selection, despite the statistical benefits of using RN models to reduce dimensionality and increase power in comparison with discrete character state approaches (Kirkpatrick and Heckman 1989; de Jong 1995; Griswold et al. 2008; Martin 2025). Mitchell and Houslay (2021), for instance, emphasize the sensitivity of inferences regarding RN slopes to the centering of intercepts. Within a given environment, the rate (slope magnitude) and direction of plastic change (slope sign) may be less relevant than the overall difference between the expressed phenotype and the local fitness optimum. Trait values for intercepts, slopes, and residuals may also be regulated by common mechanisms across environments, warranting attention to context‐specific selection on phenotypic expression rather than the selection of distinct RN parameters across contexts (Via 1993). However, as emphasized above, there is a diverse array of phenomena for which distinct genetic and physiological mechanisms have been identified as regulators of variation in trait plasticity per se, independently of variation in mean phenotypes (Scheiner and Lyman 1991; Suzuki and Nijhout 2006; Projecto‐Garcia et al. 2017; Ørsted et al. 2018; Futuyma 2021). There are also many studies demonstrating the potential for direct selection on and the evolution of generalized mechanisms of plasticity, facilitating RN‐based prediction of phenotypic expression beyond the environmental contexts over which selection previously occurred. Research on learning processes provides many elegant examples (Wright et al. 2022; Leadbeater and Thornton 2025). The work of Liefting et al. (2018) in wasps (*Nasonia vitripennis*), for instance, demonstrated that selection on context‐specific conditioned responses to visual stimuli led to the evolution of generalized associative learning ability independently of learning context, reward, or stimulus modality. This body of research supports the value of treating RNs as a biologically meaningful target of selection.

Ultimately, empiricists should leverage phenotype‐ and system‐specific considerations when motivating a causal interpretation of selection gradients estimated from our model, in addition to the necessary quantitative and experimental considerations required for causal inference (Pearl et al. 2016). In other cases, it may be more sensible to treat the model as a pragmatic means of more efficiently and unbiasedly estimating selection on different components of individual variation. In general, complex patterns of nonlinear fluctuating selection on the phenotype, which are very challenging to study empirically, can be much more easily detected and estimated with respect to the attendant selection effects expected on RN parameters (de Jong 1995; Martin, Westneat, Nakagawa, et al. 2025). Therefore, regardless of theoretical interpretation, the proposed modeling framework should prove useful for enhancing empirical tests of adaptive theory on a variety of labile traits in the wild.

## Author Contributions

**Jordan S. Martin:** conceptualization (lead), formal analysis (lead), funding acquisition (lead), investigation (lead), methodology (lead), software (lead), validation (lead), visualization (lead), writing – original draft (lead), writing – review and editing (lead). **Yimen G. Araya‐Ajoy:** conceptualization (supporting), funding acquisition (supporting), methodology (supporting), supervision (supporting), writing – review and editing (supporting). **Niels J. Dingemanse:** conceptualization (supporting), funding acquisition (supporting), methodology (supporting), supervision (supporting), writing – review and editing (supporting). **Alastair J. Wilson:** conceptualization (supporting), funding acquisition (supporting), methodology (supporting), supervision (supporting), writing – review and editing (supporting). **David F. Westneat:** conceptualization (supporting), funding acquisition (supporting), methodology (supporting), supervision (supporting), writing – review and editing (supporting).

## Conflicts of Interest

The authors declare no conflicts of interest.

## Supporting information


**Appendix S1:** ece372298‐sup‐0001‐AppendixS1.pdf.

## Data Availability

R and Stan code with detailed tutorials for implementing the models presented in this paper are available online through a GitHub public repository https://github.com/jordan‐scott‐martin/selection‐on‐rns as well as Zenodo (https://doi.org/10.5281/zenodo.17367926).
