# Supplementary material for: Estimating (Non)Linear Selection on Reaction Norms: A General Framework for Labile Traits
Source: Ecol Evol. 2025 Oct 24;15(10):e72298. doi: 10.1002/ece3.72298 (PMC12550410; doi:10.1002/ece3.72298)
Supplement: Supplementary file 1 — Appendix S1: ece372298‐sup‐0001‐AppendixS1.pdf. [file ECE3-15-e72298-s001.pdf]

Supplementary material for

Estimating (non)linear selection on reaction norms:

A general framework for labile traits

Jordan S. Martin*[1], Yimen G. Araya-Ajoy[2], Niels J. Dingemanse[3],
Alastair J. Wilson[4], & David F. Westneat[5]

*corresponding author: jordanscott.martin@eawag.ch

Contents

Code Repository S2
Repeatable among-individual differences due to RNs S2
Estimating selection gradients S3
  Standardizing selection gradients S4
Model extensions and generalizations S4
  Adjusted and nonlinear effects S4
  Additional individual effects S6
  Fluctuating selection S7
  Multivariate generalization S8
References S11

Code Repository

R and Stan code to assist in application of the proposed models can be found both on Github (https://github.com/Jordan-Scott-Martin/Selection-on-RNs) as well as Zenodo (https://doi.org/10.5281/zenodo.17367926). See the `nls-tutorial.pdf` file to get started.

Repeatable among-individual differences due to RNs

Selection on the reaction norms (RNs) of labile traits can only occur if individuals differ in their intercepts, slopes, and/or residual parameters across time. The covariance matrix $\boldsymbol{P}$ in **Eq. 1** describes these repeatable among-individual differences and, therefore, ultimately determines the total amount of trait (co)variation available to natural selection on phenotype $\boldsymbol{z}$ over the sampling period of interest, given that RN parameters $\boldsymbol{\mu_0}$, $\boldsymbol{\beta_x}$, and $\boldsymbol{\sigma_0}$ predict how organisms will repeatedly express their phenotype within and across environments. We denote the total magnitude of repeatable among-individual differences in $\mathbf{z}$ due to RNs as $\mathrm{var}(\boldsymbol{\eta})$, which in the general case sets an upper limit on the heritability of a phenotype due to direct genetic effects (see Bijma, 2011 for social traits) and thus provides a useful phenotypic proxy of the evolvability of a trait (Martin et al., 2023). The trait values $\boldsymbol{\eta}$ represent the repeatable character states that organisms are expected to express within and across sampled environments, as predicted by their RNs (**Figure 1**). Conversely, any variance in observed trait values $\mathbf{z}$ due to non-repeatable effects $\mathrm{var}(\boldsymbol{\xi}) = \mathrm{var}(\boldsymbol{z}) - \mathrm{var}(\boldsymbol{\eta})$ introduces noise into the estimation of selection gradients defined across sampled environments. Failure to distinguish non-repeatable $\mathrm{var}(\boldsymbol{\xi})$ and repeatable $\mathrm{var}(\boldsymbol{\eta})$ variance in measured phenotypes $\mathrm{var}(\mathbf{z}) = \mathrm{var}(\boldsymbol{\eta}) + \mathrm{var}(\boldsymbol{\xi})$ can thus lead to biased estimates of directional $\boldsymbol{\beta}^*$ and quadratic $\boldsymbol{\gamma}^*$ selection gradients (**Figure 2**). For evolutionary ecologists, correlations between fitness and phenotype that are repeatable over time and potentially heritable across generations will generally be of primary interest, motivating partitioning of $\mathrm{var}(\boldsymbol{\eta})$ from $\mathrm{var}(\mathbf{z})$ with a GLMM (Martin & Jaeggi 2022).

O'Dea et al. (2022) and de Villemereuil et al. (2016), among others, provide exact analytic solutions and numeric methods for calculating $\mathrm{var}(\boldsymbol{\eta})$ with many

commonly used GLMMs. For the general case, however, $\mathrm{var}(\boldsymbol{\eta})$ can always be approximated on the original data scale, irrespective of model complexity, by using simulation to compare the variance of model predicted phenotypic distributions in the presence $\mathrm{var}(\boldsymbol{z}_{\mathrm{pred}})_{\boldsymbol{\eta}}$ and absence $\mathrm{var}(\boldsymbol{z}_{\mathrm{pred}})_{-\boldsymbol{\eta}}$ of repeatable individual effects $\boldsymbol{\mu_0}$, $\boldsymbol{\beta_x}$, and $\boldsymbol{\sigma_0}$, using a large number of random samples.

$$\mathrm{var}(\boldsymbol{\eta}) \approx \mathrm{var}(\boldsymbol{z}_{\mathrm{pred}})_{\boldsymbol{\eta}} - \mathrm{var}(\boldsymbol{z}_{\mathrm{pred}})_{-\boldsymbol{\eta}} \qquad (\mathbf{S1})$$

Model predictions can also be used to approximate the total repeatability of among-individual differences in the phenotype on the original data scale for any GLMM

$$\mathrm{R}_\eta \approx \frac{\mathrm{var}(\boldsymbol{\eta})}{\mathrm{var}(\boldsymbol{z}_{\mathrm{pred}})_{\boldsymbol{\eta}}} \qquad (\mathbf{S2})$$

The bias of estimated selection gradients will increase as the R_η of a phenotype decreases and $\mathrm{var}(\boldsymbol{\xi})$ in turn increases (Spearman, 1904; Searle, 1961). Therefore, failure to remove non-repeatable causes of variation from observed phenotypic measures is particularly problematic when estimating selection on labile traits across heterogeneous environments (**Figure 2**; Dingemanse et al. 2021).

Estimating selection gradients

Following Lande and Arnold (1983) and Morrissey and Sakrejda (2013), directional $\boldsymbol{\beta}$ and quadratic $\boldsymbol{\gamma}$ selection gradients can be numerically calculated for any GLMM by taking the first ∂ and second ∂^2 partial derivatives of the estimated fitness function with respect to the expected population-level RN parameters μ_0, β_x, and σ_0.

$$\beta_{\mu_0} = \frac{\partial \bar{W}}{\partial \mu_0} \bar{W}^{-1} \dots \; \gamma_{\mu_0} = \frac{\partial^2 \bar{W}}{\partial \mu_0^2} \bar{W}^{-1} \dots \; \gamma_{\beta_x \sigma_0} = \frac{\partial^2 \bar{W}}{\partial \beta_x \, \partial \sigma_0} \bar{W}^{-1} \qquad (\mathbf{S3})$$

where $\bar{W}$ is the expected population fitness on the original data scale, as predicted by the fitness function defined with $\boldsymbol{b}$ and $\boldsymbol{q}$ coefficients on the link scale in **Eq. 4**. The directional gradients β_{μ_0}, β_{β_x}, and β_{σ_0} indicate the direction and magnitude of selection on the expected values of population RN parameters, with respect to the original untransformed scale of the data. Quadratic selection gradients γ_{μ_0}, γ_{β_x} and γ_{σ_0} in turn indicate convex or concave curvature in the selection surface shaping the variance of

RN parameters (Stinchcombe et al. 2008); and $\gamma_{\mu_0\beta_x}$, $\gamma_{\mu_0\sigma_0}$, and $\gamma_{\beta_x\sigma_0}$ indicate further curvature due to the presence of correlational selection between RN parameters (Blows & Brooks 2003). Example R code for numerically calculating these derivatives using model estimates can be found in the associated repository for the paper.

Standardizing selection gradients

Standardized gradients are particularly useful for GLMMs because the magnitude of variances may differ appreciably between the distinct transformed link scales used for estimating RNs and selection, which makes it challenging to meaningfully distinguish between small and large effect sizes across models. Selection gradients can be expressed in standardized units for effect size comparison between traits and parameters using the appropriate variances and standard deviations (Lande & Arnold 1983)

$$\beta_{\mu_0}^{\mathrm{sd}} = \beta_{\mu_0}\,\mathrm{sd}(\boldsymbol{\mu_0}) \;\ldots\; \gamma_{\mu_0}^{\mathrm{sd}} = \gamma_{\mu_0}\mathrm{var}(\boldsymbol{\mu_0}) \ldots\; \gamma_{\beta_x\sigma_0}^{\mathrm{sd}} = \gamma_{\beta_x\sigma_0}\mathrm{sd}(\boldsymbol{\beta_x})\mathrm{sd}(\boldsymbol{\sigma_0}) \qquad (\mathbf{S4})$$

or, when appropriate for application (Hereford et al., 2004), population mean values

$$\beta_{\mu_0}^{\mathrm{m}} = \beta_{\mu_0}\,\mu_0 \;\ldots\; \gamma_{\mu_0}^{\mathrm{m}} = \gamma_{\mu_0}\mu_0^2 \;\ldots\; \gamma_{\beta_x\sigma_0}^{\mathrm{m}} = \gamma_{\beta_x\sigma_0}\beta_x\,\sigma_0 \qquad (\mathbf{S5})$$

Model extensions and generalizations

Simplified models are presented in the main text (**Eq. 1-2**) to aid interpretation, but it will often be necessary to specify more complex models for explaining empirically observed variation in fitness and phenotype. Various model extensions can be straightforwardly accomplished using the basic toolkit of GLMMs and related regression frameworks, along with appropriate study design and sufficient repeated sampling for reliable estimation. Below we briefly consider three key areas for model extension and provide references for further consideration. Implementation for social traits and interactions is discussed by Martin and Jaeggi (2022).

Adjusted and nonlinear effects

As with any regression analysis, additional fixed and random effects may need to be adjusted for to facilitate appropriate biological inference. Predation may, for instance, cause differential mortality as a function of repeatable differences in behavior

across sex and age classes, but this selection will not generate an evolutionary response on behavioral variation within sexes or age classes. This motivates estimating repeatable individual variation adjusted for the effects of sex and age, among other commonly studied factors such as size and morphology (Bolnick et al., 2003). Unadjusted environmental effects on fitness and phenotype can also bias estimates of selection and among-individual variation in both field and laboratory settings (Scheiner et al. 2002; Stinchcombe et al., 2002; Kinsler et al., 2023; Munar-Delgado et al., 2023). It is, therefore, often useful to include additional environmental covariates (e.g. average temperature and rainfall, date within season, resource availability), as well as potential interaction effects, and random factors (e.g. nesting site, spatial position, batch, observer identity) to adjust fitness variation during the selection analysis. As discussed above, model predictions can always be used to quantify and better understand how adjusting for these effects changes the repeatable variation available to selection in any multivariate GLMM.

Relationships between fitness, phenotype, and the local environment may also be best described by additional terms beyond quadratic regression coefficients. For example, RN slopes of thermoregulatory and life history traits such as growth rate are often highly nonlinear in response to temperature (Oomen & Hutchings, 2022), violating the assumption that individuals' phenotypic deviations from the linear RN slope β_x are multivariate normally distributed. Polynomials or generalized additive effects such as splines and Gaussian processes (Schluter & Nychka, 1994; Sigourney, Munch, & Letcher, 2012; Catalina, Bürkner, & Vehtari, 2020) can be used to account for nonlinearity in the population RN and ensure the statistical model more accurately predicts observable phenotypic and fitness variation. In the general case, the basic model (**Eq. 2**) can be expanded to include any generalized additive function *s*() describing how expected phenotypic μ_{jt} or fitness values θ_{jt} change in response to the environment

$$g_\mu(\mu_{jt}) = \mu_0 + \mu_{0j} + s(x_{jt}) + \beta_{xj}x_{jt} \qquad \textbf{(S6)}$$

$$g_\theta(\theta_{jt}) = W_0 + W_{0j} + s(x_{jt}) + b_1\mu_{0j} + b_2\beta_{x_j} + b_3\sigma_{0j} \ldots$$

Extensive tutorials for incorporating such nonlinear effects into Bayesian regression models in Stan are freely available online (see https://mc-

stan.org/users/documentation/case-studies for worked examples of fitting splines and Gaussian processes). Code from Stan models constructed using familiar R syntax in the brms package (Bürkner, 2018) can also provide a helpful reference point for getting started. By allowing for arbitrarily complex average RN shapes across subjects, individual deviations $\boldsymbol{\beta}_x$ from the average slope for phenotype as well as for fitness are much more likely to exhibit multivariate normality. This general approach allows researchers to accurately describe trait change across complex and dynamic environments, while still using standard theory from quantitative genetics to quantify selection gradients and predict short-term evolutionary responses.

Additional individual effects

The RN model presented in the main text (**Eq. 1-2**) does not account the fact that phenotypic dispersion σ may also be plastic across environments, a phenomenon broadly referred to as 'malleability' (see O'Dea, Noble, & Nakagawa 2021 for discussion). Malleability in residuals can be estimated by including population- and individual-level slopes in the linear predictor of the dispersion parameter (Westneat et al., 2013). For example,

$$g_\sigma(\sigma_{jt}) = \sigma_0 + \sigma_{0j} + (\rho + \rho_j)x_{jt} \qquad (\mathbf{S7})$$

where observation-level variation in environmental measure **x** is expected to have effect ρ on average differences in phenotypic residuals. Malleability can then be treated as a further RN parameter that is also potentially under selection. Some statistical distributions such as the Poisson lack an explicit dispersion parameter, due to deterministic mean-variance relationships, and thus at first glance only provide scope for selection on the RN intercepts and slopes of expected values. However, in many empirical datasets, there is more variance observed in the phenotype than predicted by these distributions (overdispersion), which can be accounted for through the inclusion of further random effects capturing stochastic, observation-level deviations from model expectations (i.e. residuals; Harrison, 2014). Taking the same approach described in **Eq. 1-2**, the dispersion of these observation-level random effects can then be modelled as a function of individual-level intercepts and slopes, similar to a standard Gaussian model, providing scope for estimating selection on phenotypic variability across a broader range of RN GLMMs.

More generally, any theoretically relevant component of a statistical distribution may be modelled as a function of further individual-level effects and conceptualized as a RN parameter regulating the expression of phenotypes within and across environments. Hurdle models, for example, combine multiple distributions together to distinguish effects on the presence/absence of trait expression from effects on the subsequent magnitude or intensity of trait expression (Mullahy 1986; Heilbron 1994). This is particularly useful for phenotypes such as allogrooming behavior in primates, which can vary repeatably among individuals both in its probability of occurring as well as its intensity and duration once expressed (Silk et al., 2017). These processes are interdependent but may nonetheless be subject to distinct selection pressures (e.g. whom should be groomed and how much), which can be investigated by estimating separate RN intercepts and/or slopes on both model components.

Fluctuating selection

Fluctuating selection on RNs may occur due to variation in the density of mates and competitors, resource availability and seasonality, bodily condition and age, the availability of local niches, or any other state that modulate the fitness costs and benefits of labile traits (Houston & McNamara, 1999; Sih et al., 2015). Fluctuating selection is also expected to be a key mechanism for explaining patterns of macroevolutionary stasis (Estes & Arnold, 2007), the adaptive evolution of phenotypic plasticity (de Jong, 1995; King & Hadfield, 2019; Martin et al., 2025), and the evolutionary maintenance of individual and genetic variation within populations more generally (e.g. Sasaki & Ellner, 1997; Dingemanse & Wolf, 2010; Wolf & Weissing, 2010; Wright et al., 2019; Abdul-Rahman, Tranchina, & Gresham, 2021; Martin et al., 2023). Quantitative genetic theory has demonstrated the mathematical equivalence of models for selection on character states and RNs. A key finding from this theoretical work is that fluctuating selection on character states expressed within environments generates directional and quadratic selection on RN parameters across environments (Gavrilets & Scheiner, 1993; de Jong, 1995; Martin et al., 2025). Therefore, estimating non-zero directional and quadratic selection on a RN parameter implies that selection on the phenotype is fluctuating with respect to the environment over which the RN parameter is defined (de Jong, 1995; Martin et al., 2025). For example, the degree to which density-dependent selection on character states fluctuates across the environments encountered by a population is expected to be proportional the

directional and quadratic selection on the RN slope β_x defining phenotypic change with respect to density *x*. In general, this means that the RN selection model can be used to infer the presence of fluctuating phenotypic selection using many fewer parameters than an equivalent character state model.

These considerations suggest that RN selection analyses will often not require estimating additional parameters beyond the main linear ***b*** and nonlinear ***q*** effects on RN parameters to accurately describe patterns of fluctuating selection on the expressed phenotype. However, in the presence of environmental change, the magnitude and pattern of fluctuating character state selection experienced by a population may also vary across space and time, which is expected to result in fluctuating selection on RN parameters (**Figure 2**). In some systems, the putative environmental causes of fluctuating selection will be directly measured, while in others, it may be informative to estimate spatiotemporal heterogeneity in RN selection even if the underlying causes are not directly measured (Reynolds, de Los Campos, Egan, & Ott 2016). For example, long-term field studies can be used to investigate the adaptive maintenance of RN variation by yearly fluctuations in selection, even if the mechanisms underpinning these fluctuations remain unclear (de Villemereuil et al., 2020; Mouchet et al., 2021). To incorporate such effects, the basic fitness model (**Eq. 2**) can be extended by including fixed or random interaction effects on the selection coefficients, which will estimate continuous or discrete fluctuations in selection gradients $\boldsymbol{\Delta\beta}$ and $\boldsymbol{\Delta\gamma}$ across space and time. For example,

$$g_\theta(\theta_{jt}) = W_0 + W_{0j} + (b_1 + b_{1x}x_{jt} + u_{tb_1})\mu_{0j} + \cdots (q_1 + q_{1x}x_{jt} + u_{tq_1})\mu_{0j}^2 + \cdots \quad \text{(S8)}$$

where b_{1x} and q_{1x} describe how the (non)linear selection coefficients change as a function of x_{jt}, and u_{tb_1} and u_{tq_1} describe changes due to a random factor at time *t*.

Multivariate generalization

The models presented in the main text (**Eq. 1-2**) are intentionally simplified for conceptual clarity. However, the (non)linear selection model can be straightforwardly extended to RNs of any desired complexity, as well as to multiple potential phenotypes under selection as well as multiple fitness components. Using matrix notation, a generalized multivariate nonlinear selection model for RNs can be given by

$$z_{jtp} \sim f\left(\mu_{jtp}, \sigma_{jtp}\right) \qquad \textbf{(S9.1)}$$

$$g_{\mu_p}\left(\mu_{jtp}\right) = \boldsymbol{X}_{\boldsymbol{\mu_{jtp}}}\boldsymbol{\beta}_{\boldsymbol{\mu_p}} + \boldsymbol{Y}_{\boldsymbol{\mu_{jtp}}}\boldsymbol{u}_{\boldsymbol{\mu_{jp}}}$$

$$g_{\sigma_p}\left(\sigma_{jtp}\right) = \boldsymbol{X}_{\boldsymbol{\sigma_{jtp}}}\boldsymbol{\beta}_{\boldsymbol{\sigma_p}} + \boldsymbol{Y}_{\boldsymbol{\sigma_{jtp}}}\boldsymbol{u}_{\boldsymbol{\sigma_{jp}}}$$

$$\boldsymbol{u_j} = \left[\boldsymbol{u}_{\boldsymbol{\mu_{1j}}}^{\top}, \boldsymbol{u}_{\boldsymbol{\sigma_{1j}}}^{\top}, \dots, \boldsymbol{u}_{\boldsymbol{\sigma_{Pj}}}^{\top}\right]^{\top} \sim \mathrm{MVN}(\mathbf{0}, \mathbf{P})$$

$$W_{jtc} \sim f\left(\theta_{jtc}, \delta_c\right)$$

$$g_{\theta_c}\left(\theta_{jtc}\right) = \boldsymbol{X}_{\boldsymbol{\theta_{jtc}}}\boldsymbol{\beta}_{\boldsymbol{\theta_c}} + W_{0cj} + \boldsymbol{b}_{\boldsymbol{c}}^{\top}\boldsymbol{u_j} + \boldsymbol{u}_{\boldsymbol{j}}^{\top}\boldsymbol{Q_c}\boldsymbol{u_j}$$

$$\left[W_{0_1}, \dots, W_{0_{Cj}}\right]^{\top} \sim \mathrm{MVN}(\mathbf{0}, \mathbf{P})$$

Where $\boldsymbol{z_p}$ is an observed value for trait $p = 1, \dots, P$, and $\boldsymbol{W_c}$ is a fitness measure for component $c = 1, \dots, C$. For each trait, there are fixed $\boldsymbol{X\beta}$ effects and $\boldsymbol{Yu}$ random effects on the expectations μ and residual trait scales σ for individual *j*. The matrices $\boldsymbol{X}$ and $\boldsymbol{Y}$ are the respective fixed and random design matrices subset for the corresponding values of individual *j* at time *t* (note that we use $\boldsymbol{Y}$ to avoid confusion with notation for phenotypes and fitness), and $\boldsymbol{\beta}$ and $\boldsymbol{u}$ are vectors containing the respective fixed and individual random effect sizes. The multivariate normally distributed individual random effects for all RN parameters across all traits $\boldsymbol{u}_{\boldsymbol{\mu_{1j}}}, \boldsymbol{u}_{\boldsymbol{\sigma_{1j}}}, \dots, \boldsymbol{u}_{\boldsymbol{\sigma_{Pj}}}$ are organized into a vector $\boldsymbol{u_j}$ for individual *j*. Here $\top$ indicates transpose so that $\boldsymbol{u_j}$ is a column vector. This vector enters the fitness function for quantifying selection effects. For the expectation θ of each fitness component *c* for observation *t* of individual *j*, there are component-specific linear $\boldsymbol{b_c}$ and quadratic $\boldsymbol{Q_c}$ selection coefficients (note that $\boldsymbol{Q_c}$ is simply a matrix containing $\boldsymbol{q_c}$), as well as individual-level random deviations W_{0cj} capturing unexplained selection effects. Of course, further random effects at other hierarchical levels (e.g. groups, nesting sites, years of observation) can also be included for both the fitness and phenotype model but are ignored here for clarity (see prior subsections). The fitness model residual scale could also be modelled as a function of predictors. As noted in the main text and above, the parameterization for quadratic effects is given here in traditional statistical fashion, without the $\frac{1}{2}$ scaling factor outside of $\boldsymbol{Q_c}$ as in the Lande & Arnold (1983)

model, and thus requires further scaling to quantify quadratic selection gradients (see *estimating selection gradients* above).

To incorporate fluctuating selection, the fitness model can be further modified with effect size vectors $\boldsymbol{\Delta}_{\mathbf{b}_d}$ and matrices $\boldsymbol{\Delta}_{\mathbf{Q}_d}$ for each predictor *d* in an $\boldsymbol{F}$ matrix of fixed and/or random predictors (**Eq. S8**). These effects capture proportional fluctuations in the selection gradients $\boldsymbol{\Delta\beta}$ and $\boldsymbol{\Delta\gamma}$ (**Fig. 2**) across environments, after appropriate transformation.

$$g_{\theta_c}(\theta_{jtc}) = \boldsymbol{X}_{\boldsymbol{\theta_{jtc}}}\boldsymbol{\beta}_{\boldsymbol{\theta_c}} + W_{0cj} + \boldsymbol{b}_{\boldsymbol{cjt}}^{\top}\boldsymbol{u}_{\boldsymbol{j}} + \boldsymbol{u}_{\boldsymbol{j}}^{\top}\boldsymbol{Q}_{\boldsymbol{cjt}}\boldsymbol{u}_{\boldsymbol{j}} \qquad (\mathbf{S9.2})$$

$$\boldsymbol{b}_{\boldsymbol{cjt}} = \boldsymbol{b}_{\boldsymbol{c0}} + \sum_{d}^{D} F_{jtd}\, \boldsymbol{\Delta}_{\mathbf{b}_{\boldsymbol{d}}}$$

$$\boldsymbol{Q}_{\boldsymbol{cjt}} = \boldsymbol{Q}_{\boldsymbol{c0}} + \sum_{d}^{D} F_{jtd}\, \boldsymbol{\Delta}_{\mathbf{Q}_{\boldsymbol{d}}}$$

The coefficients in $\boldsymbol{b}_{\boldsymbol{c0}}$ and $\boldsymbol{Q}_{\boldsymbol{c0}}$ represent the expected selection effects when $\boldsymbol{F} = \mathbf{0}.$

This presentation is less intuitive but important for emphasizing that the models in **Eq. 1-2** are a simple example of what is possible more broadly within this framework. The key innovation we have proposed is the simultaneous estimation of random individual effects on traits $\boldsymbol{u}$ and their selection effects $\boldsymbol{b}$ and $\boldsymbol{q}$ (or equivalently, $\boldsymbol{Q}$) on fitness components, which as shown by **Eq. S9**, can in principle be accomplished for models of any complexity. The dimensionality of **Eq. S9** can grow rapidly with many RN parameters, phenotypes, and fitness components, making it challenging to accurately estimate a fully parameterized model. Our simulations also suggest that power for estimating parameter-specific selection gradients is heavily impacted by the integration of RN components across individuals (**Fig. 4**). Therefore, as with high-dimensional phenotypes more generally, researchers should consider how dimension-reduction techniques can be incorporated into the analysis without substantial sacrifice of biological interpretation. Techniques such as principal component and structural equation modeling can, for instance, be incorporated into the model for reducing multiple RN parameters to a smaller subset of latent variables. Alternatively, high-dimensional models can also be investigated using heavily penalizing priors such as the regularized horseshoe to effectively sort and identify stronger signals of selection (Pirronen & Vehtari, 2017), as well as to promote more principled strategies for model reduction with techniques such as projection predictive feature selection (Catalina et

al., 2020; McLatchie et al., 2025; https://mc-stan.org/projpred/articles/projpred.html for computational details).

References

Abdul-Rahman, F., Tranchina, D., & Gresham, D. (2021). Fluctuating environments maintain genetic diversity through neutral fitness effects and balancing selection. *Molecular Biology and Evolution*, *38*(10), 4362–4375.

Bijma, P. (2011). A general definition of the heritable variation that determines the potential of a population to respond to selection. *Genetics*, *189*(4), 1347–1359.

Blows, M. W., & Brooks, R. (2003). Measuring nonlinear selection. *The American Naturalist*, *162*, 815–820.

Bolnick, D. I., Svanbäck, R., Fordyce, J. A., Yang, L. H., Davis, J. M., Hulsey, C. D., & Forister, M. L. (2003). The ecology of individuals: incidence and implications of individual specialization. *The American Naturalist*, *161*(1), 1–28.

Bürkner, P. (2018). Advanced Bayesian multilevel modeling with the R package brms. *The R Journal*, *10*, 395–411.

Catalina, A., Bürkner, P. C., & Vehtari, A. (2020). Projection predictive inference for generalized linear and additive multilevel models. *arXiv*. http://arxiv.org/abs/2010.06994

Dingemanse, N. J., Araya-Ajoy, Y. G., & Westneat, D. F. (2021). Most published selection gradients are underestimated: Why this is and how to fix it. *Evolution*, *75*, 806-818.

Dingemanse, N. J., & Wolf, M. (2010). Recent models for adaptive personality differences: a review. *Philosophical Transactions of the Royal Society B: Biological Sciences*, *365*, 3947-3958.

Estes, S., & Arnold, S. J. (2007). Resolving the paradox of stasis: models with stabilizing selection explain evolutionary divergence on all timescales. *The American Naturalist*, *169*, 227–244.

Gavrilets, S., & Scheiner, S. M. (1993). The genetics of phenotypic plasticity. VI. Theoretical predictions for directional selection. *Journal of Evolutionary Biology*, *6*, 49–68.

Harrison, X. A. (2014). Using observation-level random effects to model overdispersion in count data in ecology and evolution. *PeerJ*, *2*, e616.

Heilbron, D. C. (1994). Zero-altered and other regression models for count data with added zeros. *Biometrical Journal*, *36*, 531–547.

Hereford, J., Hansen, T. F., & Houle, D. (2004). Comparing strengths of directional selection: How strong is strong? Evolution 58, 2133–2143.

Houston, A. I., & McNamara, J. M. (1999) *Models of adaptive behaviour*. Cambridge, MA: Cambridge University Press.

de Jong, G. (1995). Phenotypic plasticity as a product of selection in a variable environment. *The American Naturalist*, *145*, 493–512.

King, J. G., & Hadfield, J. D. (2019). The evolution of phenotypic plasticity when environments fluctuate in time and space. *Evolution Letters*, *3*, 15–27.

Kinsler, G., Schmidlin, K., Newell, D., Eder, R., Apodaca, S., Lam, G., Petrov, D., & Geiler-Samerotte, K. (2023). Extreme sensitivity of fitness to environmental conditions: Lessons from #1BigBatch. *Journal of Molecular Evolution*, *91*, 293–310.

Lande, R., & Arnold, S. J. (1983). The measurement of selection on correlated characters. *Evolution*, *37*, 1210–1226.

McLatchie, Y., Rögnvaldsson, S., Weber, F., & Vehtari, A. (2025). Advances in projection predictive inference. *Statistical Science*, *40*(1), 128–147.

Martin, J. S., & Jaeggi, A. V. (2022). Social animal models for quantifying plasticity, assortment, and selection on interacting phenotypes. *Journal of Evolutionary Biology*, *35*, 520-538.

Martin, J. S., Jaeggi, A. V., & Koski, S. E. (2023). Social evolution of individual differences: Future directions for a comparative science of personality in social behavior. *Neuroscience & BioBehavioral Reviews*, *144*, 104980.

Martin, J. S., Westneat, D. F., Wilson, A. J., Dingemanse, N. J., & Araya-Ajoy, Y. (2025). Frequency-dependence favors social plasticity and facilitates socio-eco-evolutionary feedback in fluctuating environments. *Functional Ecology*. Doi: **10.1111/1365-2435.70132**

Morrissey, M. B., & Sakrejda, K. (2013). Unification of regression-based methods for the analysis of natural selection. *Evolution*, *67*, 2094–2100.

Mouchet, A., Cole, E. F., Matthysen, E., Nicolaus, M., Quinn, J. L., Roth, A. M., Tinbergen, J. M., van Oers, K., van Overveld, T., & Dingemanse, N. J. (2021). Heterogeneous selection on exploration behavior within and among West European populations of a passerine bird. *Proceedings of the National Academy of Sciences*, *118*.

Mullahy, J. (1986). Specification and testing of some modified count data models. *Journal of Econometrics*, *33*, 341–365.

Munar-Delgado, G., Araya-Ajoy, Y. G., & Edelaar, P. (2023). Estimation of additive genetic variance when there are gene–environment correlations: Pitfalls, solutions and unexplored questions. *Methods in Ecology and Evolution*, *14*, 1245-1258.

O'Dea, R. E., Noble, D. W., & Nakagawa, S. (2021). Unifying individual differences in personality, predictability and plasticity: A practical guide. *Methods in Ecology and Evolution*, *13*, 278-293.

Oomen, R. A., & Hutchings, J. A. (2022). Genomic reaction norms inform predictions of plastic and adaptive responses to climate change. *The Journal of Animal Ecology*, *91*, 1073–1087.

Piironen, J., & Vehtari, A. (2017). Sparsity information and regularization in the horseshoe and other shrinkage priors. *Electronic Journal of Statistics*, *11*(2), 5018–5051.

Reynolds, R. J., de Los Campos, G., Egan, S. P., & Ott, J. R. (2016). Modelling heterogeneity among fitness functions using random regression. *Methods in Ecology and Evolution*, *7*, 70–79.

Sasaki, A., & Ellner, S. (1997). Quantitative genetic variance maintained by fluctuating selection with overlapping generations: Variance components and covariances. *Evolution*, *51*, 682–696.

Scheiner, S. M., Donohue, K., Mazer, L. A. D. S. J., & Wolfe, L. M. (2002). Reducing environmental bias when measuring natural selection. *Evolution*, *56*, 2156–2167.

Schluter, D., & Nychka, D. (1994). Exploring fitness surfaces. *The American Naturalist*, *143*, 597–616.

Searle, S. R. (1961). Phenotypic, genetic and environmental correlations. *Biometrics*, *17*, 474–480.

Sigourney, D. B., Munch, S. B., & Letcher, B. H. (2012). Combining a Bayesian nonparametric method with a hierarchical framework to estimate individual and temporal variation in growth. *Ecological Modelling*, *247*, 125–134.

Sih, A., Mathot, K. J., Moirón, M., Montiglio, P. O., Wolf, M., & Dingemanse, N. J. (2015). Animal personality and state–behaviour feedbacks: A review and guide for empiricists. *Trends in Ecology and Evolution*, *30*, 50–60.

Silk, J. B., Roberts, E. R., Barrett, B. J., Patterson, S. K., & Strum, S. C. (2017). Female–male relationships influence the form of female–female relationships in olive baboons, Papio anubis. *Animal Behaviour*, *131*, 89–98.

Spearman, C. (1904). The proof and measurement of association between two things. *The American Journal of Psychology*, *15*, 72–101.

Stinchcombe, J. R., Rutter, M. T., Burdick, D. S., Tiffin, P., Rausher, M. D., & Mauricio, R. (2002). Testing for environmentally induced bias in phenotypic estimates of natural selection: theory and practice. *The American Naturalist*, *160*, 511–523.

Stinchcombe, J. R., Agrawal, A. F., Hohenlohe, P. A., Arnold, S. J., & Blows, M. W. (2008). Estimating nonlinear selection gradients using quadratic regression coefficients: Double or nothing? *Evolution*, *68,* 2435-2440.

de Villemereuil, P., Charmantier, A., Arlt, D., Bize, P., Brekke, P., Brouwer, L.,..., & Chevin, L. M. (2020). Fluctuating optimum and temporally variable selection on

breeding date in birds and mammals. *Proceedings of the National Academy of Sciences*, *117*, 31969–31978.

Villemereuil, P. de, Schielzeth, H., Nakagawa, S., & Morrissey, M. (2016). General methods for evolutionary quantitative genetic inference from generalized mixed models. *Genetics*, *204*, 1281–1294.

Westneat, D. F., Schofield, M., & Wright, J. (2013). Parental behavior exhibits among-individual variance, plasticity, and heterogeneous residual variance. *Behavioral Ecology*, 24, 598-604.

Wolf, M., & Weissing, F. J. (2010). An explanatory framework for adaptive personality differences. *Philosophical Transactions of the Royal Society B*, *365*, 3959–3968.

Wright, J., Bolstad, G. H., Araya-Ajoy, Y. G., & Dingemanse, N. J. (2019). Life-history evolution under fluctuating density-dependent selection and the adaptive alignment of pace-of-life syndromes. *Biological Reviews*, *94*, 230–247.
